# Intratesticular Epidermoid Cyst: A Rare Tumor

**DOI:** 10.4021/jocmr474w

**Published:** 2010-12-11

**Authors:** Jose Aneiros-Fernandez, Salvador Arias-Santiago, Barbara Cancela-Diez, Francisco O'Valle, Jose Aneiros Cachaza

**Affiliations:** a Department of Pathology, University Hospital, Granada, Spain; b Department of Pharmacology, University Hospital, Granada, Spain

## Abstract

Testicular epidermoid cyst is extremely rare injury, which in recent studies has been regarded as a tumor. Some authors consider this lesion as a cystic monodermal teratoma, the histogenesis remains controversial. In this study we provide clinical and morphological characteristics of an epidermoid cyst in the left testicle in a 16 years old patient, where apparently a nodular lesion in the left testicle was detected.

**Keywords:**

Cyst; Epidermoid; Intratesticular

## Introduction

Intratesticular epidermoid cysts are infrequent tumors that usually appear in young, still very rare in pediatric age, which account for 1% of all testicular tumors. The clinical diagnosis of this tumor poses a major challenge because the clinical course is not different as how it behaves as a malignant tumor [1]. Ultrasound examination may facilitate the diagnosis because it has an onion-skin pattern, and laboratory data revealed normal in tumor markers.

We present an unusual case in a 16 years old male with intratesticular epidermoid cyst.

## Case Report

A 16 years old male had a lesion of nodular appearance in the left testicle. The ultrasound shows a hypoechoic lesion, with the analytical and negative markers. Suspecting a tumor, left orchiectomy was performed. Pathology refers to a piece of left orchiectomy of 5.5 cm in length, presenting a cystic soft white content of 1.5 × 1.2 cm in diameter without affecting the epididymis or tunica albuginea.

**Figure 1.**
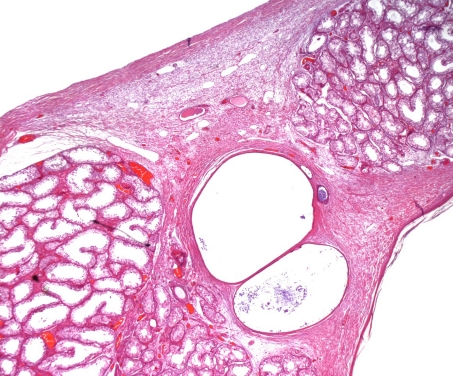
Shows epidermoid cyst adjacent to a germinal epithelium of the tubules that retained the normal characteristics (Magnification × 4, hematoxylin and eosin).

**Figure 2.**
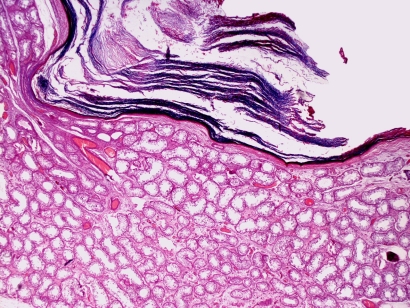
Histological section showing intraparenchymal cyst with a squamous epithelium with laminated keratin (Magnification × 20, hematoxylin and eosin).

The histopathological study demonstrated a cystic structure lined by a multilayered squamous epithelium, without evidence of atypical changes (Fig. 1, 2). Serial study was performed which demonstrated no pilosebaceous structures or the presence of endodermal and mesodermal components. It also noted some cavities in the vicinity of the mesothelium lining the albuginea. These cavities are lined by a row of cells, which correspond to interpret invaginations of the mesothelium. Cavities are also evident in isolation similar to those previously mentioned, material containing keratin. The rest of the testis showed seminiferous tubules with changes in the maturation of the germinal series without evidence of intratubular neoplasia.

**Figure 3.**
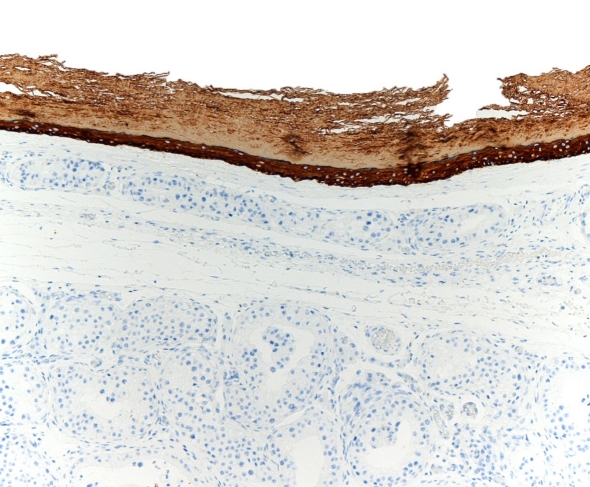
Immunohistochemical staining shows positivity with 34BE12 investment material cyst epithelium.

Immunohistochemistry was performed on intratesticular epidermoid cyst, showing positive for high molecular weight CK (CK 5-6 and 34bE12) in all of them (Fig. 3) with low positive basal Ki-67. The spaces in proximity to the mesothelium expressed CK5-6, CAM 5.2, CK 8 and 34bE12 as the mesothelium.

## Discussion

Epidermoid cysts are benign lesions that often are located in the skin and are very rare in intratesticular level. At the histological level, they are composed of the same characteristics as those of the skin location. They are solitary lesions in the onset age of 20 - 40 years in white males [1]. Although in children, very rare cases have been published and even bilateral.

We considered several histogenetic possibilities for intratesticular epidermoid cyst, such as a source at the expense of metaplasia of the seminiferous tubules or the rete testis; other molecular biology studies consider that in some cases corresponding to a tumor as a cystic monodermal teratoma of less origin has been frequently suggested in the scrotum infundibular cysts [3, 4].

Clinically, lesions appear as firm, well circumscribed, small, solitary, painless, which are sometimes indistinguishable from malignant tumors.

There are tools to make a correct diagnosis: (a) absence of the analytical elevation of tumor markers such as alpha fetoprotein and beta-HCG; (b) the ultrasound finding that is very characteristic of this disease, which comprises hyperechoic heterogeneous sonographic pattern which gives the appearance of 'onion skin', accompanied by a peripheral hypoechoic zone; (c) magnetic resonance imaging typically shows the image in 'bull's eye', which consists of a low intensity center because it is composed of dense calcifications and remains, a high-intensity mid-zone which consists of scaly squamous cells, and other peripheral zone of low intensity in T1 and T2 due to compact keratin fibers; (d) with no Doppler vascularization [5, 6].

The histologic diagnosis of epidermoid cyst was described by Price in 1969, a study of 69 cases, 5 therefore established criteria: (a) the injury must be located within the testicular parenchyma; (b) the center cyst composition should be amorphous material keratinized debris; (c) within the cyst, teratoid elements or components of dermal appendages should not exist; (d) there should be no scars in the parenchyma adjacent to the epidermoid cyst [7].

The treatment of choice for intratesticular epidermoid cyst is excision of the cyst with the surrounding testicular parenchyma to check no teratomatous component or malignant germ cell neoplasia accompanying. Postoperative monitoring is recommended [8].

## References

[1] Aguilera Tubet C, Lopez Rasines G, Roca Edreira A, Martin Garcia B, Hernandez Rodriguez R, Portillo Martin JA, Gutierrez Banos JL (2005). [Testicular epidermoid cyst: uncommon lesion of difficult preoperative diagnosis]. Actas Urol Esp.

[2] Sloan JC, Beck SD, Bihrle R, Foster RS (2002). Bilateral testicular epidermoid cysts managed by partial orchiectomy. J Urol.

[3] Reinberg Y, Manivel JC, Llerena J, Niehans G, Fraley EE (1990). Epidermoid cyst (monodermal teratoma) of the testis. Br J Urol.

[4] Younger C, Ulbright TM, Zhang S, Billings SD, Cummings OW, Foster RS, Eble JN (2003). Molecular evidence supporting the neoplastic nature of some epidermoid cysts of the testis. Arch Pathol Lab Med.

[5] Langer JE, Ramchandani P, Siegelman ES, Banner MP (1999). Epidermoid cysts of the testicle: sonographic and MR imaging features. AJR Am J Roentgenol.

[6] Ching-Yang C, Ching-Jiunn W, Wei-Chiung L (2005). Unusual MR imagings of an incidental testicular epidermoid cyst: A case report. Chin J Radiol.

[7] Price EB (1969). Epidermoid cysts of the testis: a clinical and pathologic analysis of 69 cases from the testicular tumor registry. J Urol.

[8] Heidenreich A, Engelmann UH, Vietsch HV, Derschum W (1995). Organ preserving surgery in testicular epidermoid cysts. J Urol.

